# Emergence of Serotype G12 Rotaviruses, Hungary

**DOI:** 10.3201/eid1306.061181

**Published:** 2007-06

**Authors:** Krisztián Bányai, Ágnes Bogdán, Péter Kisfali, Péter Molnár, Ilona Mihály, Béla Melegh, Vito Martella, Jon R. Gentsch, György Szücs

**Affiliations:** * Baranya County Institute of State Public Health Service, Pécs, Hungary; † University of Pécs, Hungary; ‡ ”St. Laszlo” Central Hospital for Infectious Diseases, Budapest, Hungary; § University of Bari, Bari, Italy; ¶ Centers for Disease Control and Prevention, Atlanta, Georgia, USA

**Keywords:** rotavirus, G12, surveillance, genotyping, sequencing, phylogenetic analysis, dispatch

## Abstract

We describe the emergence of serotype G12 rotaviruses (67 [6.9%] of 971 specimens tested) among children hospitalized with rotavirus gastroenteritis in Hungary during 2005. These findings are consistent with recent reports of the possible global spread and increasing epidemiologic importance of these strains, which may have implications for current rotavirus vaccination strategies.

Group A rotaviruses are the leading cause of acute severe gastroenteritis in infants and young children worldwide. Approximately 130 million children are infected with rotavirus, and nearly 450,000–750,000 die of disease caused by this agent each year (*1*). Group A rotaviruses are classified into G and P serotypes and genotypes on the basis of antigenic and genetic diversity of the outer capsid proteins, VP7 and VP4, respectively. At present, 11 of 15 G types and 12 of 26 P types are known to infect humans. On a global basis, most severe infections are caused by 5 G types (G1–G4 and G9) and 3 P types (P1A[8], P1B[4], and P2A[6]), although considerable differences exist in some areas, especially in tropical countries (*2**,**3*).

One year after its introduction in 1998, RotaShield, the first licensed rotavirus vaccine, was withdrawn from use due to an association with intussusception. Recently, 2 new candidate vaccines, RotaTeq and Rotarix, were licensed for use in >40 countries and introduced into the vaccine market of several nations. RotaTeq was developed with the aim of providing serotype-specific immunity against the 4 common G types (G1–G4) and 1 common P type (P1A[8]). In contrast, Rotarix vaccine, a monovalent vaccine containing a single P1A[8],G1 strain, is expected to induce heterotypic immunity to a variety of strains that have epidemiologic and clinical importance. However, the increasing number of reports of the emergence of novel G and P types in various countries raises concerns about the adequacy of current vaccination strategies (*2**,**3*). Strain surveillance studies during and after introduction of these vaccines are needed to gauge their impact on circulating strains and monitor for possible emergence of rotavirus types that escape the immunity provided by the vaccines.

As part of the Hungarian rotavirus strain surveillance program, we obtained fecal samples from community-acquired rotavirus gastroenteritis patients <15 years of age who were admitted with acute dehydrating diarrhea in 2005 to the “St. Laszlo” Hospital, Budapest. The rotavirus-positive samples (confirmed by use of an immunochromatographic assay with Rota Uni-Strip [Coris BioConcept, Gemblaux, Belgium]) were delivered to our laboratory on a monthly basis. Routine strain characterization included polyacrylamide gel electrophoresis of the viral genome (electropherotyping) and genotyping of the outer capsid genes, VP7 and VP4, by reverse transcription–PCR (RT-PCR), as described previously (*4*). The G-typing algorithm included the use of 3 primer sets (specific for G1–G4, G6, and G9). In spite of the use of a variety of primer sets, 85 (8.7%) of the 971 analyzed specimens were nontypeable G serotypes. A relatively high number of these untypeable strains (n = 67) had identical RNA patterns (long electropherotype; data not shown), which suggested that they might belong to the same serotype. To investigate this possibility, we sequenced a 501-bp stretch of the VP7 gene (nt 79–579, amino acids [aa] 11–177) from 4 of these strains, using procedures we described elsewhere (*4*). These data showed that the 4 strains share 100% nt sequence identity to each other, >90% nt identity and 91% aa similarity to serotype G12 strains, and <78% similarity to other serotypes, a finding that strongly suggested that they belonged to serotype G12 (data not shown).

To test the remaining G-nontypeable strains, we set up a genotype-specific RT-PCR assay, using a G12-specific primer designed by Samajdar et al. (*5*), homologous to the 4 sequenced strains, in combination with a consensus VP7 gene oligonucleotide, 9Con1, to yield an amplicon of 464 bp. We first tested the specificity of this primer pair by using the Hungarian G12 strains confirmed by nucleotide sequencing and a variety of other strains (including 8 G1, 2 G2, 2 G3, 4 G4, 15 G9, and 4 untypeable strains) unrelated to the Hungarian G12 strains that were isolated during the same period. None of the non-G12 strains produced an amplicon after RT-PCR with this primer pair. In contrast, all 63 strains with the same electropherotype as the 4 sequenced G12 strains gave a 464-bp amplicon when tested with this primer pair, suggesting that they also belonged to G12. Including the 4 sequenced strains, the 67 G12 strains identified represented 6.9% of all typeable by electropheresis strains collected in the study period. Although most of the rotavirus gastroenteritis cases were identified during the peak activity of group A rotaviruses (March–April), the G12 strains also showed relative abundance in summer and autumn, representing ≈15%–28.6% of the total rotavirus strains detected during this period (Figure 1). The emergence of these strains in Hungary during 2005 raises the question of whether they were able to overcome the immunity of older children or neonates to common rotavirus strains, as demonstrated earlier when serotype G9 rotaviruses emerged in Europe. The mean and median ages of children infected with G12 rotaviruses (2.7 and 2.2 years, respectively) were, however, not significantly different from those of children infected with type G1 (3.0 and 2.5 years, respectively), G2 (2.4 and 2.0 years, respectively), G4 (2.9 and 2.4 years, respectively), and G9 (2.9 and 2.3 years, respectively) rotaviruses. Only children infected with G3 and G6 rotaviruses showed significant differences in mean and median ages compared with those for children with G12 rotavirus (G3: 4.8 and 3.8 years, respectively, p = 0.009; G6: 5.3 and 4.3 years, respectively, p = 0.047). A subset of G12 samples subjected to P genotyping (n = 14) tested positive for P[8]; this result was confirmed by sequencing results for 3 strains. No other RNA patterns were associated with G12 specificity.

**Figure 1:**
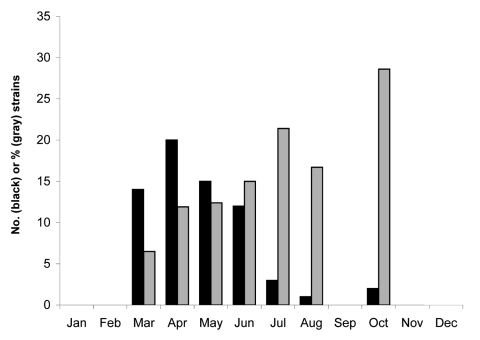
Temporal distribution of serotype G12 human rotaviruses in Budapest, Hungary, 2005. Black columns indicate the number (N) of strains identified; gray columns represent the percentage of total strains for each month that were type G1.

The first description of G12 rotaviruses dates back to the 1990s, when 2 unusual human strains (L26 and L27) from the Philippines were characterized serologically and by nucleotide sequencing as P[4],G12 with subgroup I specificity and a long electropherotype (*6**,**7*). The origin of these strains was obscure because they were apparently very rare in humans over the next 10 years and had not been previously detected in animals. Beginning in 2002, reports of the detection and increased prevalence of G12 strains have appeared from Asia (Thailand, India, Korea, Japan, Bangladesh, Nepal, and Saudi Arabia) and the Americas (the United States, Argentina, and Brazil) (*5**,**8**–**14*), including strains with as yet unpublished G12 VP7 sequences in the GenBank database. Although these strains are closely related to each other (>96.9% nt identity), they have diverged substantially from early human G12 isolates (<91% nt identity).

While the origin of G12 strains is unknown, a G12 rotavirus was recently identified in a pig, representing a possible animal source of this serotype (*15*). However, this single porcine isolate was not highly related to any known human G12 isolate (<92% nt identity), a finding that leaves gaps in our knowledge about the source of the modern lineages of serotype G12 rotaviruses from humans.

The global emergence of rotavirus G12 shares several features with the worldwide spread of serotype G9 in the mid to late 1990s (*2**,**3*). First, both serotypes were identified in humans ≈2 decades ago and subsequently were rarely detected for many years despite intensified surveillance activity and the introduction of sensitive and specific molecular typing methods. Second, sequence analysis and serologic studies demonstrated substantial genetic and antigenic differences between early and modern variants within both serotypes, which suggested a new or separate introduction into humans. Third, of these coexisting genetic lineages, only 1, the modern lineage of each serotype, has been recognized as being capable of spreading globally. Four, various associated P types have been detected and display geographic differences in their distribution. For example, G12 strains in South America and far eastern Asia contain the globally rare P[9]; the only identified G12 strain in the United States bears the regionally common P[6]; Hungarian strains are associated with the most common genotype, P[8]; and Indian strains have been found in association with globally or regionally distributed P[4], P[6], or P[8] specificities. It will be interesting to determine, whether, as with the spread of G9 strains (*3**)*, emergence of G12 rotavirus will occur predominantly through reassortment of a single gene, VP7, into a background of globally common human Wa genogroup strains, as suggested by our finding of G12 in Hungarian P[8] strains with a typical long electropherotype. Last, identification of clearly related VP7 genes in pigs suggests that we should continue to search for possible ancestors for both serotypes in the porcine species. This finding also reaffirms that pigs act as reservoirs of newly recognized human rotavirus antigenic types.

At the time of manuscript preparation, ≈40 G12 VP7 sequences were available in the DNA databases. Most sequence data have been published from India, where G12 strains showing minor genetic variation in their VP7 gene sequences (<3% nt difference) were isolated between 2001 and 2005 in various geographic regions. In contrast, all 4 Hungarian G12 strains share 100% sequence identity over a stretch of 501 nt. This finding suggests that G12 rotaviruses circulated for some years in India before their identification. By contrast, the G12 rotavirus detected in 2005 in Hungary is likely the result of a very recent introduction. One possibility is the importation of a single G12 strain into Hungary, which subsequently spread in the population. Although few data are available on the sequence of P[8] VP4 genes of G12 strains, we found that the P[8] VP4 of the Hungarian G12 strains was most closely related to the VP4 of a Saudi Arabian strain, MD844 (>99.4% nt similarity along with corresponding sequence length), confirming the epidemiologic linkage among these strains that was suggested by the nearly complete sequence identity for the VP7 gene (>99.4%, Figure 2).

**Figure 2:**
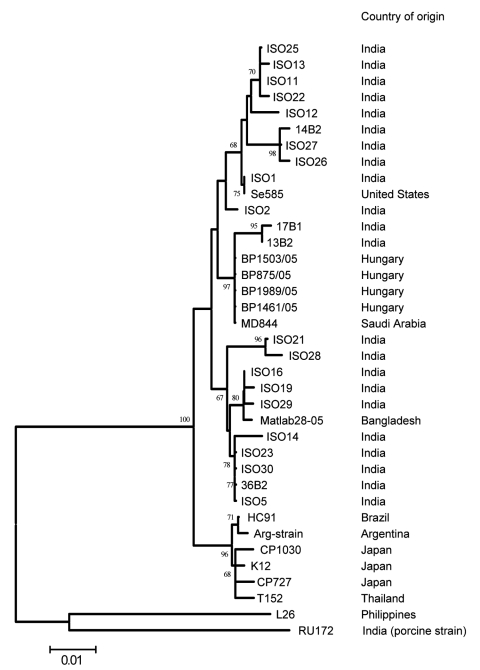
Phylogenetic relationship among Hungarian and other G12 rotaviruses. The tree was generated by the neighbor-joining algorithm by using a 501-nt fragment of VP7 (nt 79–579). Scale bar represents the nucleotide distance. Bootstrap values >60% are shown in the branch nodes. The country of origin is shown parallel to the strain names. ARG strain is an unnamed G12 isolate from Argentina**.**

Detailed molecular characterization of the entire genome is needed to help determine the extent of genetic variation and the relatedness of these Hungarian G12 strains to other, recently emerged G12 rotaviruses. More intensified investigation of animal rotavirus strains may identify the possible animal ancestor of the new genetic lineage. Continuous monitoring of human rotavirus strains circulating in local communities will be important to determine if we are again detecting the early stages of the global emergence of a novel genetic lineage of an “old” rotavirus serotype. This was seen several years ago for the global spread of a modern lineage of serotype G9 rotaviruses. This finding may have important implications for vaccine use in planned or already launched rotavirus immunization programs in numerous countries worldwide.

## References

[1] Parashar UD, Gibson CJ, Bresee JS, Glass RI. Rotavirus and severe childhood diarrhea. Emerg Infect Dis. 2006;12:304–6. 16494759 10.3201/eid1202.050006 PMC3373114

[2] Santos N, Hoshino Y. Global distribution of rotavirus serotypes/genotypes and its implication for the development and implementation of an effective rotavirus vaccine. Rev Med Virol. 2005;15:29–56. 10.1002/rmv.448 15484186

[3] Gentsch JR, Laird AR, Bielfelt B, Griffin DD, Bányai K, Ramachandran M, Serotype diversity and reassortment between human and animal rotavirus strains: implications for rotavirus vaccine programs. J Infect Dis. 2005;192:S146–59. 10.1086/431499 16088798

[4] Bányai K, Gentsch JR, Schipp R, Jakab F, Meleg E, Mihály I, Dominating prevalence of P[8],G1 and P[8],G9 rotavirus strains among children admitted to hospital between 2000 and 2003 in Budapest, Hungary. J Med Virol. 2005;76:414–23. 10.1002/jmv.20372 15902709

[5] Samajdar S, Varghese V, Barman P, Ghosh S, Mitra U, Dutta P, Changing pattern of human group A rotaviruses: emergence of G12 as an important pathogen among children in eastern India. J Clin Virol. 2006;36:183–8. 10.1016/j.jcv.2006.03.006 16679056

[6] Taniguchi K, Urasawa T, Kobayashi N, Gorziglia M, Urasawa S. Nucleotide sequence of VP4 and VP7 genes of human rotaviruses with subgroup I specificity and long RNA pattern: implication for new G serotype specificity. J Virol. 1990;64:5640–4. 2170690 10.1128/jvi.64.11.5640-5644.1990 PMC248620

[7] Urasawa S, Urasawa T, Wakasugi F, Kobayashi N, Taniguchi K, Lintag IC, Presumptive seventh serotype of human rotavirus. Arch Virol. 1990;113:279–82. 10.1007/BF01316680 2171461

[8] Griffin DD, Nakagomi T, Hoshino Y, Nakagomi O, Kirkwood CD, Parashar UD, Characterization of nontypeable rotavirus strains from the United States: identification of a new rotavirus reassortant (P2A[6],G12) and rare P3[9] strains related to bovine rotaviruses. Virology. 2002;294:256–69. 10.1006/viro.2001.1333 12009867

[9] Pongsuwanna Y, Guntapong R, Chiwakul M, Tacharoenmuang R, Onvimala N, Wakuda M, Detection of a human rotavirus with G12 and P[9] specificity in Thailand. J Clin Microbiol. 2002;40:1390–4. 10.1128/JCM.40.4.1390-1394.2002 11923362 PMC140366

[10] Das S, Varghese V, Chaudhury S, Barman P, Mahapatra S, Kojima K, Emergence of novel human group A rotavirus G12 strains in India. J Clin Microbiol. 2003;41:2760–2. 10.1128/JCM.41.6.2760-2762.2003 12791925 PMC156500

[11] Shinozaki K, Okada M, Nagashima S, Kaiho I, Taniguchi K. Characterization of human rotavirus strains with G12 and P[9] detected in Japan. J Med Virol. 2004;73:612–6. 10.1002/jmv.20134 15221908

[12] Pietruchinski E, Benati F, Lauretti F, Kisielius J, Ueda M, Volotao EM, Rotavirus diarrhea in children and adults in a southern city of Brazil in 2003: distribution of G/P types and finding of a rare G12 strain. J Med Virol. 2006;78:1241–9. 10.1002/jmv.20686 16847962

[13] Castello AA, Arguelles MH, Rota RP, Olthoff A, Jiang B, Glass RI, Molecular epidemiology of group A rotavirus diarrhea among children in Buenos Aires, Argentina, from 1999 to 2003 and emergence of the infrequent genotype G12. J Clin Microbiol. 2006;44:2046–50. 10.1128/JCM.02436-05 16757596 PMC1489448

[14] Uchida R, Pandey BD, Sherchand JB, Ahmed K, Yokoo M, Nakagomi T, Molecular epidemiology of rotavirus diarrhea among children and adults in Nepal: detection of G12 strains with P[6] or P[8] and a G11P[25] strain. J Clin Microbiol. 2006;44:3499–505. 10.1128/JCM.01089-06 17021073 PMC1594765

[15] Ghosh S, Varghese V, Samajdar S, Bhattacharya SK, Kobayashi N, Naik TN. Molecular characterization of a porcine group A rotavirus strain with G12 genotype specificity. Arch Virol. 2006;151:1329–44. 10.1007/s00705-005-0714-7 16502286

